# Editorial: Antibiotic resistance in aquatic systems, volume II

**DOI:** 10.3389/fmicb.2023.1298681

**Published:** 2023-10-23

**Authors:** Satoru Suzuki, Amy Pruden, Marko Virta, Tong Zhang

**Affiliations:** ^1^Graduate School of Science and Engineering, Center for Marine Environmental Studies, Ehime University, Matsuyama, Japan; ^2^Department of Civil and Environmental Engineering, Virginia Tech, Blacksburg, VA, United States; ^3^Department of Microbiology, University of Helsinki, Helsinki, Finland; ^4^Department of Civil Engineering, The University of Hong Kong, Hong Kong, China

**Keywords:** antibiotic resistance, aquatic environment, monitoring, low- and middle-income countries (LMICs), reservoir, culture-dependent, metagenome

Worldwide dissemination of antibiotic-resistant pathogens and antibiotic resistance genes (ARGs) is one of the most serious global health threats. Since the “One Health” concept was launched by the G8 Science Ministry in 2013 and the Elmau summit in 2015 (https://www.consilium.europa.eu/media/35254/01_2015-06-08-leaders-statement_final_clean.pdf), recognition of the need to understand the origin and ecology of antibiotic resistance has expanded across disciplines, beyond human and animal clinics and into various realms of environmental research.

In particular, the aquatic systems, including lakes, rivers, streams, marine environments, and coastlines, receive effluent from wastewater treatment plants (WWTP), runoff from agricultural activity, aquaculture, and other human inputs. Such aquatic environments can serve both as a natural reservoir of antibiotic resistance and a conduit for the spread of clinical resistance traits (Michael et al., 2013). In the Frontiers Research Topic “*Antibiotic resistance in aquatic systems*”, published in 2017, three chapters included articles on reservoirs, horizontal gene transfer, and amplification of resistance (Suzuki et al., 2017). Amarasiri et al. (2020) reviewed recent knowledge regarding antibiotic-resistant bacteria (ARB) and ARGs in aquatic environments and highlighted pervasive questions that were yet to be answered to better forecast the health risks caused by ARB and ARGs in water environments.

Advanced metagenome approaches are now being widely applied to ecological research of ARB and ARGs, expanding global risk assessment (Zhang et al., 2022) and opening avenues toward comprehensive and quantitative molecular surveillance of antimicrobial resistance. Available approaches for monitoring antibiotic resistance in the environment have their advantages but also blind spots. Thus, culture-based approaches maintain an essential role, especially when combined with whole genome and metagenomic sequencing, in advancing comprehensive and global knowledge on ARB/ARG ecology. Combining approaches and extracting quantitative information is especially valuable for endeavors such as discovering new ARB/ARGs, shedding light on hot spots for dissemination, and informing efforts to mitigate antibiotic contamination and contain ARG reservoirs. Coordinated efforts are underway to standardize methods for antimicrobial resistance monitoring, employing tiered approaches, including bacteria culture, PCR, and metagenomics, to support accessibility in low- and middle-income countries (LMICs) (Pruden et al., 2021). Integrated surveillance efforts should harmonize existing efforts and provide overall value to clinicians, veterinarians, policy-makers, and regulators. Data sharing will be particularly essential for advancing the fundamental understanding needed to combat antimicrobial resistance. In particular, regional data are needed for the control and regulation of ARB/ARG contamination through WWTP and industries in each country. Thus, each region or country can contribute toward defining appropriate approaches and targets for monitoring and reporting. Routine monitoring will provide essential data for assessing trends.

In this Research Topic, “*Antibiotic resistance in aquatic systems, volume II*”, we see much progress in countries across the globe, including Croatia, Ecuador, the Republic of Korea, China, Nepal, and Japan. Environmental monitoring of ARGs in these countries has generally been lacking. Studies included in this Research Topic employ both bacterial culture and molecular detection from environmental DNA (eDNA).

Kvesić et al. reported on WWTP submarine effluent in Croatia. *Escherichia coli, Enterobacter, Serratia, Citrobacter*, and other bacterial genera were isolated on chromogenic agar. KPC-producing *E. coli* was detected in marine water with *bla*_KPC−2_ and *bla*_OXA−48_. The escape of such highly resistant bacteria to the sea is identified as a concern. The case in Ecuador showed ESBL-*E. coli* was isolated from irrigation water, fruits, and vegetables (Montero et al.; Montero et al., 2021). Among 165 *E. coli* isolates, 58% were of the ESBL phenotype. Irrigation water showed a higher occurrence than vegetables and fruits. *bla*_CTX−M−55_, *bla*_CTX−M65_, and *bla*_CTX−M15_ were frequently found among the isolates. Detection of ARB/ARGs from Korean WWTP was performed by culture method and SmartChip-based culture-independent methods (Shin et al.). Multidrug-resistant bacteria were mainly *Citrobacter, Escherichia-Shigella*, and *Stenotrophomonas*, while *van*C, *bla*_OXA_, and *bla*_NDM_ persisted through the WWTP process. Yang et al. focused on *Legionella pneumophila* in environmental water and soil. Although they mainly examined resistance mechanisms, the persistence of *L. pneumophila* resistant to various antibiotics was found in environmental water and soil. Two research papers demonstrated eDNA analysis from Nepal and Japan. Amarasiri et al. quantitated ARGs by droplet digital PCR. River water and shallow-dug well water that are used for drinking, bathing, and other household activities were contaminated with ARGs. *sul1, intI1*, and *tet*A were abundant, and *bla*_KPC_, *bla*_OXA_, and *van*A were also detected. More than 70% of hospitals in Nepal do not have WWTP facilities, thus clinical ARGs tend to be released into rivers and might come back as a health risk to humans. In river and pond water in Tokyo, eDNA frequently contained *bla*-genes, multidrug efflux pumps, and fluoroquinolone resistance genes (Nishikawa et al.). OXA-group was abundant followed by CTX-group among the *bla* genes. In isolated multi-drug resistant bacteria, multidrug efflux genes were more abundant than specific drug ARGs, suggesting that released ARBs from clinical settings and environmental ARBs might have different resistance traits.

As editors of this Research Topic, we make a few key observations from the articles included in this Research Topic and other recent advances in ARB/ARG research. The first is the urgent need for monitoring of ARB/ARGs in clinical settings and affected water environments in LMICs. These countries tend to be in a position where they are most vulnerable to antibiotic resistance, especially where humans, livestock, and wildlife live in close proximity. The second point is the need to establish coordinated and standardized monitoring methodologies among countries. Culture-dependent methods, PCR-based quantitative methods for eDNA, and metagenome analysis for eDNA and isolated bacteria all provide value. Although some will not be feasible in some areas depending on the availability of consumables and facilities, consensus on common protocols and techniques should provide decision milestones globally. Recent articles also highlighted these challenges in this context (Pruden et al., 2021; Zhang et al., 2022).

Importantly, LMICs should not be expected to bear the brunt of the antibiotic resistance problem alone. Antimicrobials, many of them manufactured in LMICs, are used all over the world for various essential and non-essential purposes. Yet, antimicrobials are often not available when needed in LMICs, or those that are made available are not subject to sound stewardship. At the same time, water sanitation and hygiene as well as wastewater treatment are all too often insufficient or limited in these countries (Segura et al., 2015), which creates opportunities for ARB/ARGs to develop and be released into the environment (Suzuki and Hoa, 2012). The establishment of monitoring protocols in each area or country is needed to support locally appropriate strategies for mitigating the environmental dimension of ARB/ARGs. For example, *intI1* is a promising indicator gene for comprehensively quantifying anthropogenic inputs of antibiotic resistance to the environment (Gillings et al., 2015; Zhen et al., 2020). Articles in this Research Topic provide examples of monitoring ARGs in aquatic environments and the distribution and diversity of ARGs in various countries. These research papers expand our knowledge and understanding of aquatic systems as reservoirs and reactors of antimicrobial resistance development and dissemination and provide a benchmark for comparison for future studies.

## Author contributions

SS: Conceptualization, Writing—original draft. AP: Writing—review and editing. MV: Writing—review and editing. TZ: Writing—review and editing.
